# The complement system of the goat: Haemolytic assays and isolation of major proteins

**DOI:** 10.1186/1746-6148-8-91

**Published:** 2012-06-26

**Authors:** Isabel Moreno-Indias, Alister W Dodds, Anastasio Argüello, Noemi Castro, Robert B Sim

**Affiliations:** 1 Animal Science Department, Universidad de las Palmas de Gran Canaria, Arucas, Las Palmas, 35413, Spain; 2 MRC Immunochemistry Unit, Department of Biochemistry, University of Oxford, South Parks Road, Oxford, OX1 3QU, UK

**Keywords:** Goat, Complement system, C3, C1q, Factor H

## Abstract

**Background:**

The aim of the present study was to develop a haemolytic assay for the study of the complement system in dairy goats (*Capra aegagrus hircus*) and to characterize the major goat complement system proteins.

**Results:**

The commonly used sheep erythrocyte sensitized with rabbit antibodies were not sensitive to lysis by goat serum, but the combination of human red blood cells (RBC) plus rabbit antibodies was the best option found for goat complement assay. A buffer based on HEPES instead of the classical veronal (barbitone) was developed. Three proteins were isolated: factor H, C1q and C3 and these were compared with the corresponding human proteins. A novel affinity chromatography technique was developed for isolation of factor H.

**Conclusions:**

Human RBC plus rabbit antibodies were a suitable option for haemolytic assays. The isolated proteins are similar to the human counterparts.

## Background

Complement is a central component of the innate immune system which is involved in host defense against infectious agents [1]. It has three physiological activities: defending against pyogenic bacterial infection, bridging innate and adaptive immunity, and disposing of immune complexes and the products of inflammatory injury [2]. The complement system may be activated by three different pathways: the classical, the lectin and the alternative pathway [3]. The classical pathway is activated by binding of the C1q protein to targets, such as Gram negative bacteria, immunoglobulins bound to microorganisms or altered host components, such as amyloids or apoptotic cells; the alternative pathway may be directly activated by microorganisms and also by IgG immune complexes. The lectin pathway is activated by MBL (mannose-binding lectin) bound to carbohydrates present on the surface of microbes [4]. The lectin pathway of complement can also be activated *via* L-ficolin [5], H-ficolin [6], M-ficolin [7] and by the recently-described Collectin 11 [8]. The complement system in mammals has been well described, particularly in human and mice. Some other vertebrate species have been examined at various levels of detail (*e.g.* chimpanzee, dog, horse, sheep, guinea-pig, pig, cattle, chicken and some fish), and it is clear their complement systems are very similar [1]. In vertebrate studied, the complement system consists of about 35–40 proteins commonly associated with blood plasma and blood cells, but found generally at lower concentration in other secretions of the body [9], like lymph, colostrum or milk [3].

In farm animals, the complement system has been studied principally in cows [10-12], although there are some studies in other ruminants, like buffalos [9]. There is scanty research papers on goats.

Some studies on conditions for assaying haemolytic complement of goat sera [13] and in particular of the alternative complement pathway [14] have been published. Other published work on goat complement includes studies of infection with some parasites like *Trypanosoma evans*i [15] or with the estimation of the molecular size of the C1 complex [16], purification of C1q [17] or mapping of MHC-linked complement genes [18]. In theses studies, C2 and C4 were found to be MHC-linked, as in humans.

Recently, Castro *et al.*[19] demonstrated a relationship between nutrition and complement system activity in goat kids during the first two months of life. More knowledge about the complement system in goats is necessary to understand this relationship in depth.

The aim of the present study was to develop an efficient combination of antibodies and RBC to provide a feasible haemolytic assay for goat serum/plasma. An additional objective was to isolate the major complement system proteins from goat plasma, in order to raise antibodies for future quantitative concentration measurements. The proteins were C1q, the first protein involved in the classical pathway [20], factor H, because of its importance in the regulation of the alternative pathway [21], and C3, the most abundant protein (at least in humans) which forms part of all three pathways [22].

## Results

**Figure 1:**
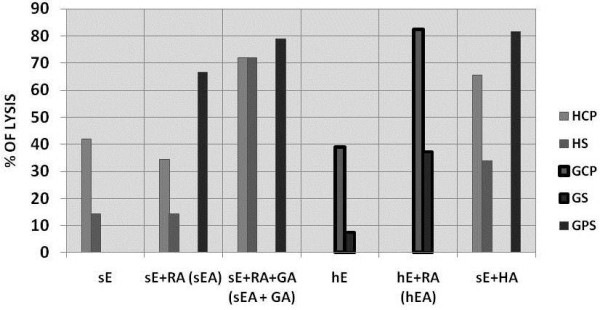
** ca="yes"Lysis of different cells, with and without antibody by human, goat and guinea-pig plasma/serum (HCP and HS, GCP and GS, and GPS, respectively).** Sheep (sE) and human (hE) erythrocytes were pre-coated or not with rabbit or rabbit + goat antibodies. Erythrocytes (50 μl of 10^9^/ml) in DGVB^++^ were incubated with 100 μl of 1/5 dilution of plasma/serum for 1 hour at 37 °C. Percentage of cell lysis was calculated as indicated in methods section.sE, hE: sheep and human erythrocytes without pre-coated; sEA, hEA = sheep and human pre-coated with rabbit antibodies; RA, GA, HA = rabbit, goat and human antibodies, respectively.

Figure 1 shows the tests results of goat, human and guinea pig serum/plasma against various RBC preparations. Goat plasma and serum showed completely different specificity from human and guinea pig serum. Goat plasma and serum lysed human RBC sensitized with rabbit antibodies, but human and guinea pig serum had no activity against these cells. There was also some lysis of unsensitised human erythrocytes by goat serum and plasma. In contrast, goat serum/plasma did not lyse sheep erythrocytes with any combination of antibodies (even with goat antibodies), while human and guinea pig sera did lyse these cells. Lysis of unsensitised sheep erythrocytes by human serum is due to the presence of anti-Forssman antibodies in the human serum/plasma.

**Figure 2:**
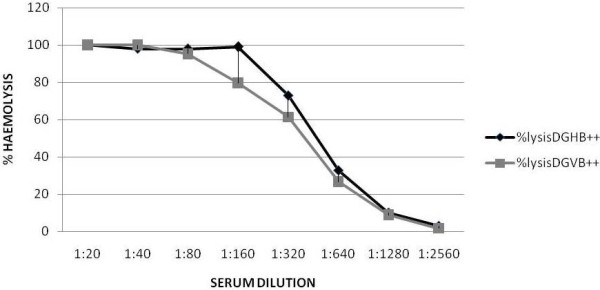
** Comparison of HEPES (DGHB**^**++**^**) and Veronal (DGVB**^**++**^**) buffers.** Serial 2-fold dilutions of human serum were prepared in either buffer, starting at 1/20 (5%). Dilutions (100 μl) were mixed with 100 μl of 10^8^/ml sheep EA cells, in the same buffer, and incubated for 1hour, at 37 °C. Percentage lysis was calculated as in methods.

It was necessary to adapt the assay to a more suitable buffer, because of the difficulty in obtaining sodium barbital (veronal) in some countries. HEPES was tried instead of barbitone. Figure 2 shows the comparison of the two buffers for an assay of human serum complement with sheep EA. Although both buffers work similarly, the titre measured was slightly higher with HEPES. Further experiences in using both buffers for goat complement showed that both are suitable for assays, but whereas DGVB^++^ is a good buffer for long-term storage of RBC, DGHB^++^ needs to be removed and changed for another buffer to store the EA cells. A good buffer to store the cells is Alsever’s Solution.

**Figure 3:**
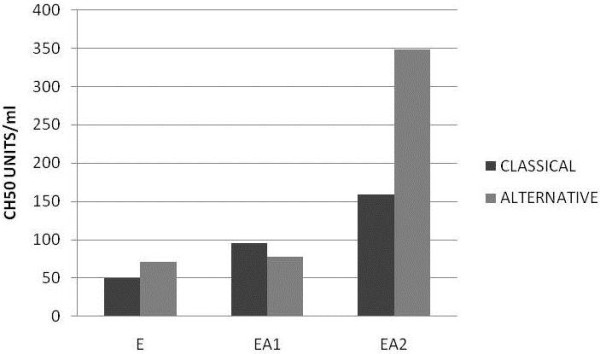
** Titration of sensitising antibody and effects of “classical pathway”*versus*“alternative pathway” condition.** Human RBC (E) were used without sensitization or sensitised with 10 μl anti(human RBC) antiserum per ml of 10^9^ cells/ml (EA1) or with 100 μl antiserum per ml of 10^9^ cells/ml (EA2). Two-fold serial dilutions of goat serum were made in either DGHB^++^ or DGHB-Mg-EGTA, starting at 1:1 (50%). Dilutions (100 μl) were incubated with E, EA1 or EA2 cells (100 μl of 10^8^/ml) in the same buffer for 1hour at 37 °C. Percentage lysis was calculated, and the serum concentration (% serum) required for 50% lysis calculated. CH_50_ (the number of complement lysis units in 100% serum).

The effects of “classical” *versus* “alternative” pathway buffers on goat complement assay are shown in Figure 3. In DGVB^++^ or DGHB^++^ all three complement system pathways can be activated, although it would be expected that the classical pathway works at lower serum concentrations than the other pathways. In DGVB-Mg-EGTA or DGHB-Mg-EGTA only the alternative pathway can work, because the other pathways require Ca^2+^ (for the binding of the proteases C1r, C1s or the MASPS, to C1q, MBL or ficolins). Two different sensitising antibody concentrations are shown. When the assay was done with hE cells, goat serum showed a titre of about 5 CH50 units in either buffer. A two-fold higher titre was obtained when the EA cells were sensitised with a low concentration of rabbit anti(human RBC) (about 80–100 CH50 units in either buffer); however, at higher antibody concentration a higher titre was observed and in the alternative pathway buffer this titre was more pronounced than in the classical pathway buffer (350 CH50 units *versus* 150 units). In a separate experiment, the concentration of antibody was varied titrating the anti-human RBC, and the maximum titre response was obtained with concentrations higher than 80 μl of antiserum per ml of cells at 10^9^/ml in DGHB++ (not shown).

**Figure 4:**
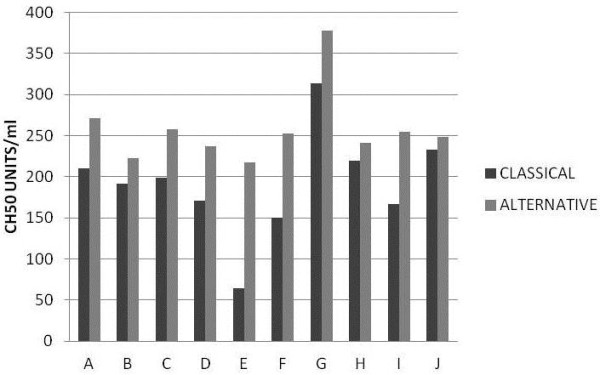
** Comparison of multiple samples.** Ten goat serum samples were assayed (as described in the legend for fig. 3) using DGHB^++^ and DGHB-Mg-EGTA buffers, and highly sensitised EA (equivalent to EA2 in fig. 3). The titre of each sample CH50 units in the assay is shown for both buffer conditions.

The fact that the titre in the alternative pathway was higher than in the classical pathway, suggests that elevated concentration of antibody present on the cells seems to activate the alternative pathway. As it has been shown by Gadd and Reid [23] antigen-bound rabbit IgG activates the alternative pathway (in human serum). The antiserum used (rabbit anti-human RBC membrane) was produced with booster injections of antigen over a 6 months period, so it had mainly an IgG response. The CH50 values for various serum samples from different goats are shown in Figure 4. These results demonstrated that this assay (using the maximum antibody sensitisation) is able to distinguish among different samples. The fact that the alternative pathway buffer, in all cases gave higher values than the classical pathway buffer is very interesting, and it must be studied in greater detail. A possible explanation could be the fact that the magnesium ion concentration affects the apparent titre in alternative pathway assays [24].

**Figure 5:**
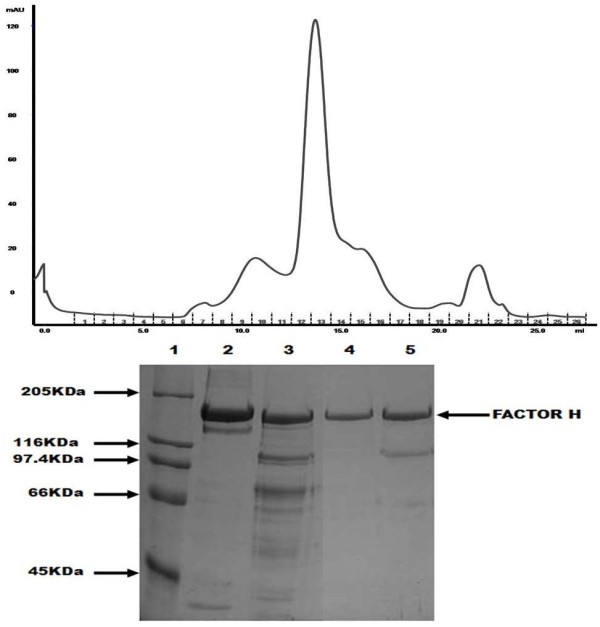
** Isolation of Goat Factor H.** a) Gel flitration of goat FH on Superose 6. b) SDS-PAGE analysis of reduced goat FH fractions. lane 1: Molecular weight standard, lane 2: human Factor H, lane 3: Factor H before the gel filtration, lane 4: fraction 12 from the gel filtration, lane 5: fraction 13 from the gel filtration. Gel was stained with Coomassie blue.

SDS-PAGE analysis of factor H (FH) purification is depicted in Figure 5. Factor H was identified by its chromatographic behaviour and co-running with human FH on SDS-PAGE; FH forms the major symmetrical peak on the final gel filtration step. As previously observed [25], human factor H binds strongly to TNP-derivatised BSA. As shown here, goat factor H also binds to TNP. This binding is of high affinity, so that high salt/denaturing buffers are required for elution of the bound protein. Factor H, however, is resistant to denaturation, and use of urea/guanidine buffers would not be expected to cause loss of activity.

**Figure 6:**
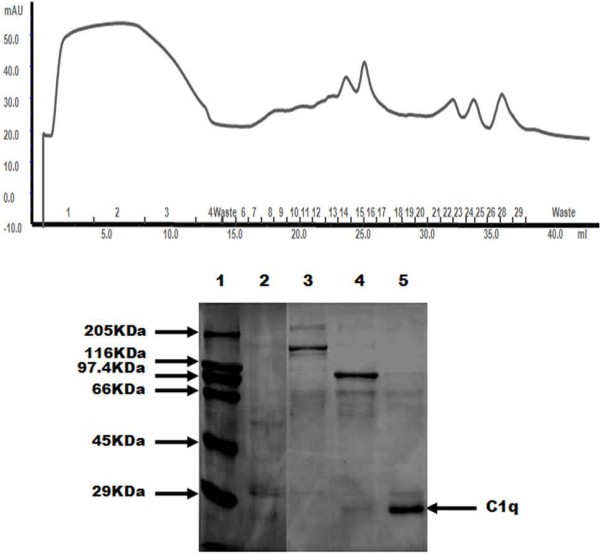
** Purification of goat C1q.** a) Ion Exchange (monoS) chromatogram of C1q. b) SDS-PAGE analysis of reduced goat C1q fractions. Lane 1: Molecular weight standard, lane 2: human C1q, (with a contaminant ~50 kDa), lane 3: fraction 16 from the ion exchange, lane 4: fraction 22, lane 5: fraction 24. Fractions were concentrated with Strataclean beads. Gel was stained with Coomassie blue.

SDS-PAGE with various fractions of goat C1q from the final mono-S ion exchange is shown in Figure 6. The yield from purification was low, but purity was adequate. Human IgG was used as the affinity ligand, and the result confirms that goat C1q does bind to human IgG, although SRBC sensitised by human IgG/IgM are not lysed by goat serum/plasma (fig 1). The Goat C1q appears similar to human C1q on SDS-PAGE. With C1q from different species, the relative elution positions of the a, b and c chains may differ. In human, a doublet band at ~ 29 kDa represents the a and b chains, while a fainter band at ~ 25kda is the c chain. The c chain aggregates in SDS-PAGE, and may also appear as faint higher bands (50–70 kDa). For goat C1q, there is a fainter band at ~ 29kda, which may represent the c chain, and a strong band at ~25 kDa, which most likely represents the co-running a and b chains. C1q is a basic high molecular weight glycoprotein which has two major problems in its purification: a low yield and contaminant inmunoglobulins [26], and because of its low yield, it was necessary to concentrate samples for SDS-PAGE analysis with Strataclean beads (Stratagene).

**Figure 7:**
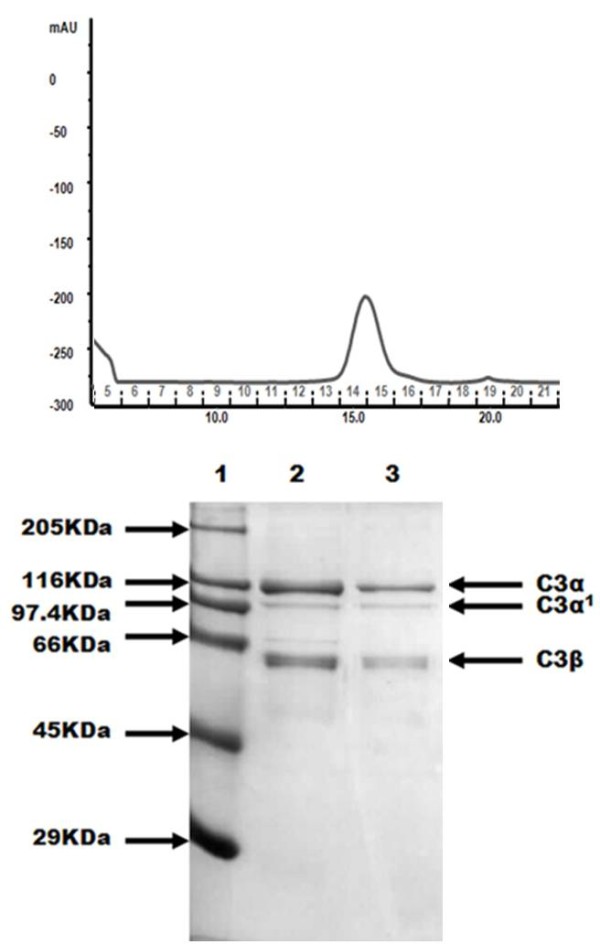
** Isolation of C3.** a) Gel filtration of goat C3 on Superose 6. b) SDS-PAGE of the final step gel filtration on Superose 6. Lane 1: Molecular weight standard, lane 2 and 3: fractions of the gel filtration (14, 15). Fractions were concentrated with Strataclean beads. Gel was stained with Coomassie blue.

SDS-PAGE analysis of fractions of the last gel filtration of the purification of C3 is shown in Figure 7. Goat C3 was of good purity although it could be contaminated with C5. C3 and C5 are homologues of the same mol. wt., and in human and rodent, they tend to co-run in chromatographic procedures [27]. The minor band under the C3 alpha chain, marked C3 alpha’, may represent either the alpha’ chain of C3b, or the alpha chain of C5.

## Discussion

Sheep erythrocytes do not work in the assay of complement in Majorera goat samples in agreement with the results of Venugopal *et al.*[14], who found negligible activity using sheep and cattle erythrocytes, working with goats of undefined breed. This was not observed previously by Olaho-Mukani *et al.*[15] who used sheep RBC with good results with African goats. Oyekan and Barta [13] showed that the best results for goat complement (breed not stated) were obtained with guinea pig or pig erythrocytes sensitized with goat or cattle antibodies, They also observed lysis with unsensitised rabbit and horse erythrocytes, and low but detectable lysis with sensitised sheep erythrocytes. Noguchi and Bronfenbrenner [28] did observe that human RBC were usable for testing goat complement. Castro and co-workers [19] used also Majorera goats for their experiment with rabbit erythrocytes without sensitizing.

The resistance to lysis of sheep RBC may be because sheep complement regulatory proteins (equivalent to CD59 or DAF) are compatible with goat complement proteins. These types of protein protect host cells from autologous complement attack [29]. These results could be due to a structural similarity between the interaction sites of proteins of both species. For a long time, bovine complement was considered to be nonhemolytic, because it was not able to lyse sheep erythrocytes [30]. However, in Holstein Bull serum, Chang–Fa *et al.*[31] did not find any relationship between phylogenetic proximity of erythrocyte species to cattle and the degree of hemolysis, attributing this response to the content of the serum of high levels of natural antibodies against RBC of various species. The highest was against guinea pigs and the lowest against cows, sheep, goats and pigs. So, it is possible that in goats something similar happens.

In general, as expected the sensitivity of the assay was improved by sensitization of the erythrocytes with specific antibodies, which enhances the activation of the classical pathway (IgG and IgM) and the alternative pathway (IgG). Buffer was changed because it is difficult to get veronal buffer in some laboratories. Veronal (sodium barbiturate) is the most widely reagent used to measure the activity of the complement system [4,13-15,32], but other buffers have been used to measure the activity. For example Castro *et al.*[19] used PBS and saline to measure the total complement system activity in kid goat serum although to measure the alternative pathway it was necessary to use the normal EGTA-Mg-gelatin-Veronal buffer. HEPES is a good buffering agent for maintaining physiological pH. The major problem was the storage of RBC in this buffer, which was much poorer than in DGVB^++^. For this reason is better to use another buffer, such as Alsever’s solution for storage, to wash the erythrocytes frequently and to minimize as much as possible the storage period in HEPES. The high values for titres in the alternative pathway buffer were unexpected. The “classical pathway” buffer allows activation of all three pathways, while the “alternative pathway” buffer allows only the alternative pathway to work.

It was expected a lower titre for the alternative pathway as the classical pathway usually dominates the activity of the complement system in most species, *e.g.* classical pathway activity is detectable in more dilute serum than is lectin or alternative pathway activity [4]. The same titre value in the two buffer systems would suggest that the activity of the complement system is due to the alternative pathway, but in our work a higher value was observed in the alternative pathway buffer. These results are consistent with previous findings showing that goats have potent alternative pathway activation as was suggested by Venugopal *et al.*[14], and supported by Castro *et al.*[19] and Rodríguez *et al.*[33] who, working with Majorera goat kids, found that the only pathway used was the alternative. The alternative pathway is continually activated at a low controlled rate but amplified by the surface of invading microorganisms [34]. The environment where the goats live is controlled, *e.g.*, nutrition, hygiene or health are supervised by the University staff, and it is thought that microbes that activate the alternative pathway are less pathogenic than those which do not [35], so that mutation causing loss of the classical pathway (*e.g.* loss of C4) might not be a survival factor for these goats.

The higher titre found in the alternative pathway buffer could be due to the higher Mg^2+^ concentration; Fishelson and Müller-Eberhard [24], showed that raising the Mg^2+^ concentration increased the alternative pathway titre. It would be interesting to probe with different of Mg^2+^concentrations, Venugopal *et al.*[14] found that higher levels of Mg^2+^ ions (>5 mM) had an inhibitory effect on the caprine alternative pathway. Matheswaran *et al.*[9], working with buffalo colostrum and with concentrations from 4 to 20 mM of Mg^2+^, found no differences, although they chose the concentration of 4 mM as optimum.

The apparent absence of classical pathway could be due to a failure in some component of the classical pathway cascade, possibly a C4 deficiency, or perhaps just because some components were in low concentration, as it occurs in bovine milk with C1q [36].

Factor H controls the activity of the alternative pathway C3/C5 convertase by competing with factor B for C3b binding and by serving as a cofactor for factor I, to mediate the cleavage of C3b to iC3b [29]. Many procedures have been used to purify factor H. In humans, Sim and Discipio [21] used five chromatography steps to isolate Factor H: first two steps of polyethyleneglycol (PEG) precipitation were used, continuing with L-Lysine- Sepharose 4B chromatography, DEAE-Sephadex A-50 chromatography, Sepharose 6B gel filtration, Hydroxyl-Apatite-Ultrogel chromatography and finally a DEAE-Sepharose CL-6B chromatography. Nakano *et al.*[37] used a shorter procedure to isolate rabbit factor H using various precipitation steps with PEG then three steps of chromatography on DEAE Sephacel; and a further two steps of Sephadex G200 gel filtration. Mhatre and Aston [38] used a first step of PEG precipitation then three steps of chromatography: DEAE-Sephacel, CM-Sephadex A-50 and Sephadex G-200 to isolate bovine Factor H. More recently, Factor H has been isolated from porcine seminal plasma by using a Q-Sepharose column then a Matrex Gel Red A column, finishing with an FPLC Superdex 200 column [39]. The method used in the present study seems to be easier and faster and with good results as shown in Figure 5. It has three steps: TNP-BSA- Sepharose, protein G Hi-Trap and Superose 6. Binding of human factorH to TNP-BSA had been observed before [24], but this is the first report where this ligand for affinity purification is used.

C1q has a critical function in host defence and clearance of immune complexes, and for this reason it is desirable to study goat C1q in more depth. Lin *et al.*[17] used 2 different methods for the purification of goat C1q: in one, the procedure was based on two successive precipitation steps at low ionic strength, and followed by an additional purification through a Sepharose CL-6B gel filtration column; and in the other, they used a two-step chromatography, with a BioRex 70 and a Sepharose CL-6B columns. C1q is a protein which has a high affinity for heparin and IgG, so McKay [40] used these characteristics to make a two-step affinity chromatography procedure to purify C1q from various species (human, rat, rabbit, dog and sheep). Pohl *et al.*[41] isolated C1q using a three-step purification procedure: precipitation from plasma, affinity chromatography with a rabbit IgG-Sepharose column and cleaning up with a rabbit anti-human IgG-Sepharose affinity column. Sasaki *et al.*[42] working with guinea pig serum, combined precipitation with chelating agents, CM-cellulose and Superose 6, and Stemmer and Loos [43] used a simple and rapid procedure for the purification of C1q from human, guinea pig and mouse serum, with euglobulin precipitation, chromatography on Superose 6B, then Mono S ion exchange.

To establish a simple method to isolate goat complement C1q, previous reports were used as references. As several procedures used immobilised IgG, non-immune human IgG-Sepharose was chosen to begin, followed by a Protein G Hi-trap column to remove contaminant human IgG, ending with ion exchange chromatography on MonoQ and MonoS to remove all the contaminants. The yield was very low, possibly because the concentration of this protein in goats is low, or because human IgG may not be a very high affinity ligand. As noted above, it is possible that C1q is present in low concentration, in that there was very low apparent classical pathway complement activation.

Although little is known about the goat complement genome, only goat C9 and Factor B have been sequenced as far as we know, a goat genome project is in progress [44] and probably in the next years the sequences of the complement system proteins will be known, so that we will be able to compare the sequences with humans or other species and learn about potential structural or functional differences.

Complement C3 may be the most studied protein of the complement system, due to its abundance and importance in all three pathways. It has been studied and isolated from many species and usually C3 has been isolated in a similar way with a first step of differential precipitation with PEG, continuing with column chromatography (*e.g.* Giclas *et al.*[45] for rabbit and Gresham *et al.*[46] for human). Storm *et al.*[47] isolated porcine C3 with PEG precipitation and DEAE-Sephacel chromatography, ending with a size exclusion chromatography with Sepharose CL-6B and a hydroxylapatite chromatography to remove the contaminants. Basta and Hammer [48], established a two step protocol to isolate C3: PEG precipitation and fast protein liquid chromatography (FPLC) Mono Q ion exchange chromatography, using human and guinea pig. A procedure used in fishes like the spotted wolfish, in which a PEG precipitation, continued by a MonoQ and a Superose 12 exclusion chromatography was described by Abelseth *et al.*, [49]. Most recent procedures for the isolation of this protein have been based on the work of Dodds [50] which was used in the present study and consists of PEG precipitation, and 2–3 ion-exchange steps.

There has been no report of C3 isolation from goats, but in other ruminants, C3 has been purified from cows [51] using a four step protocol: polyethylene glycol precipitation and chromatography on DEAE-Sephadex A-50, CM-Sephadex A-50 and Sephacryl S-200. In camels, the same procedure was followed by Ouma *et al.*[52]. In the present study the protocol has been simplified to three steps based on a Q Sepharose FF, followed by a MonoQ and a Superose 6.

## Conclusions

The alternative pathway of the goat complement system is the predominant pathway. Human RBC plus rabbit antibodies are the best option for haemolytic assays. HEPES buffer is a good alternative for laboratories where Veronal buffer is difficult to acquire. The main proteins Factor H, C1q and C3 in goats were isolated and are similar to the human counterparts.

## Materials and methods

This study was performed in accordance with adequate ethical standards and animal care and was approved by the Ethics and Animal Welfare Committee of the University of Las Palmas de Gran Canaria, Las Palmas, Spain.

### Plasma/serum

Blood samples from Majorera goats were obtained at Las Palmas de Gran Canaria University (Animal Science Department). Health status of the animals was suitable. Animals were vaccinated against *Staphylococcus aureus* and their feeding regime was based on corn, soy 66, dehydrated lucerne, dehydrated beetroot, wheat straw and a vitamin–mineral corrector, in accordance with the guidelines issued by L’Institut de Recherche Agronomique [53].

Blood was taken from the jugular vein into a tube with buffered sodium citrate 0.106 M (100 ml of this buffer to 1 L of blood) and centrifuged for 10 minutes at 2,130 g and 4 °C. Plasma was then frozen at −80 °C and transported on dry ice to Oxford University where laboratory determinations were performed. The initial sample was citrated-plasma, so it was converted to serum by adding CaCl_2_ to a final concentration of 20 mM, incubating for 20 minutes at 37 °C and centrifuging for 15 minutes at 2,500 g.

### Haemolytic assays

#### Buffers

Initial haemolytic assays were based on reagents described by Whaley and North [25]. DGVB^++^ buffer (Dextrose Gelatin Veronal Buffer, with Ca^++^ and Mg^++^:2.5 mM sodium barbital, 71 mM NaCl, 0.15 mM CaCl_2_, 0.5 mM MgCl_2_, 2.5%(w/v) glucose, 0.1% (w/v) gelatin, pH 7.4) was used for the classical pathway and DGVB-Mg-EGTA buffer (2.1 mM sodium barbital, 59 mM NaCl, 7.0 mM MgCl_2_, 2.08%(w/v) glucose, 0.08% (w/v) gelatin, 10 mM EGTA, pH 7.4) for the alternative pathway.

In later analyses the DGVB^++^ buffer was changed for DGHB^++^ buffer in which 5 mM HEPES replaced 2.5 mM sodium barbital and the DGVB-Mg-EGTA was changed for DGHB-Mg-EGTA, in which 4.2 mM HEPES replaced 2.1 mM sodium barbital

#### Preparation of antibody-sensitised erythrocytes (EA)

EA cells were prepared as described by Whaley and North [25]. Sheep erythrocytes (sE) were from sheep blood in Alsevers (TCS Biosciences Ltd., Buckinghamshire, UK) and rabbit antibody was haemolysin (Sigma-Aldrich, Poole, UK). sE and sEA were prepared as described by Whaley and North [25]. To prepare sheep erythrocytes with goat antibodies, (sEA + GA), goat-anti-rabbit IgG antibodies were added to sEA. sEA (0.5 ml at 10^9^/ml) were incubated with 0.5 ml (1:1000) goat anti-rabbit IgG (Sigma-Aldrich, Poole, UK) for 1 hour at RT. After that, two washes in DGVB^++^ were done. Human erythrocytes (hE) were prepared from blood collected from healthy volunteers, taken with EDTA as anticoagulant and rabbit anti-(human RBC membrane) antiserum was from the MRC Immunochemistry Unit. Blood samples were centrifuged (10 min, 2500 g) and plasma was removed and stored for later assays. hE were washed in PBS- 0.5 mM EDTA and then 2 times in DGHB^++^ until the supernatant was clear. Then the concentration of hE was adjusted to 10^9^/ml as for sE. To prepare hEA cells, 10 ml of hE cells were incubated with 100 μl of rabbit anti-(human RBC membrane) antiserum heat-treated for 30 minutes at 56 °C. After that, 2 washes in DGHB^++^ were done and cell number adjusted to 10^9^/ml. sE were also sensitised with human antibodies (anti-Forssman). Two ml of sE at a concentration of 10^9^/ml and 1 ml of human EDTA-plasma were mixed and incubated for 30 min at 37 °C. Then cells were washed with DGVB^++^ and adjusted again to 10^9^/ml.

#### Establishing a haemolytic assay for goat serum

Tests were done with different combinations of erythrocytes and antibodies to establish what system was most sensitive to lysis by goat complement. These combinations were: sheep RBC, sheep RBC plus rabbit antibodies (haemolysin, containing IgM and IgG), sheep RBC plus rabbit haemolysin plus goat IgG anti-rabbit IgG (Sigma), human RBC, human RBC plus rabbit antihuman red blood cell membrane (IgM and IgG) and sheep RBC plus human antibodies (IgM and IgG anti-Forssman). In initial tests, 50 μl of cells or antibody-sensitized cells at 10^9^/ml was incubated with 100 μL of serum or plasma diluted to 1/5 in DGVB^++^ for 1 hr at 37 °C before calculation of lysis as described below.

#### Haemolytic titration assay

A typical classical pathway (CH50) test was done using 100 μl of goat serum serial 2-fold dilutions (starting at 50% serum, serially diluting 10 times) in DGVB^++^, mixed with 100 μl of 10^8^/ml EA cells in DGVB^++^. E cells were also used as controls in place of EA. Cells and sera were incubated for 1 hr at 37 °C, centrifuged (10 min, 2,500 g) and the OD 405 of the supernatant measured to assess lysis. Percentage complement-dependent lysis was calculated relative to cells lysed in water (100% lysis) and cells incubated with buffer only (0% complement-dependent lysis). Alternative pathway (AP50) assays were done in the same way, but with DGVB-Mg-EGTA as buffer. In later analyses the assay buffers were DGHB^++^ or DGHB-Mg-EGTA. To calculate CH50 or AP50, % lysis was plotted against % serum and the serum dilution giving 50% lysis was determined. The CH50 or AP50 was calculated as 100/(%serum causing 50% lysis) x10 and is expressed as units per ml.

#### Factor H isolation

For the isolation of factor H a commercial serum (caprine serum, Sera Lab International Ltd) was used. It was diluted 1:1 with water to reduce ionic strength. For the isolation of the factor H, 200 ml of the goat serum dilution was mixed with 20 ml of TNP-BSA-Sepharose, prepared as indicated by Arnold *et al.*[54], for one hour in a slow rotary stirrer at 4 °C. The resin was thoroughly washed with HEPES buffer (10 mM HEPES, 60 mM NaCl, 0.5 mM EDTA pH 7.4). This affinity column binds mainly Factor H, IgG and IgM [54]. Three different solvents were assessed for elution of the bound protein: urea buffer (10 mM MES, 6 M urea pH 6.0); 2 M NaCl; and 6 M guanidine-HCl buffer (6 M guanidine-HCl, 10 mM Tris, 100 mM Na phosphate pH 8.0-8.2). The resin was placed in a column and washed successively with the three solvents and fractions of 4 ml were collected. Elution was monitored by reading OD 280. Fractions containing protein were dialysed against PBS-0.05 mM EDTA. The best eluants were the 2 M NaCl and Guanidine buffer, according to the amount of eluted protein measured at OD280 and analysis by SDS-PAGE. Fractions eluted in these solvents were pooled together and passed thought a 1 ml Protein G “Hi-Trap” column (GE Healthcare) to remove IgG. The column was washed with PBS- 0.05 M EDTA and the IgG was eluted with Glycine/HCl buffer (0.2 M glycine adjusted to pH 2.2 with HCl). To purify Factor H further gel filtration was used with a Superose 6 10/300 GL column (GE Healthcare), using filtered PBS-5 mM EDTA as running buffer. Purification was monitored by SDS-PAGE analysis using human factor H as standard.

#### C1q isolation

The diluted goat serum which passed through TNP-BSA-Sepharose was used to prepare C1q. For the isolation of subcomponent C1q, 25 ml of non-immune human IgG-Sepharose prepared as indicated by Sim *et al.*[55] was added to 200 ml of the diluted goat serum and incubated for 1 hour close to 0 °C on a slow rotary stirrer. The IgG-Sepharose was rapidly and thoroughly washed on a scintered glass filter with ice-cold PBS- 5 mM EDTA and bound proteins were eluted with ice-cold CAPS buffer (50 mM CAPS, 1 M NaCl, 5 mM EDTA, pH 11.0 to 11.2). The pH of the eluted fractions was reduced to 7.5 by adding a buffering substance (0.5 M NaH_2_PO4). The eluted protein (without dialysis) was passed through a Hi-Trap protein G column to remove IgG. The protein solution was then dialysed against 10 mM HEPES, 10 mM NaCl pH7.4, and run on a MonoQ 5/5 column (GE Healthcare) equilibrated in the same buffer. C1q does not bind to the column, but contaminants were removed. The run-through fraction containing C1q was finally dialysed against 230 mM Na-acetate at pH 5.2 and run on a monoS 5/5 column (GE Healthcare) in the same buffer. C1q was eluted in a linear gradient of NaCl concentration (0–400 mM NaCl in the acetate buffer). Purification was monitored by SDS-PAGE analysis using human C1q as standard.

#### C3 isolation

For isolation of protein C3, citrated plasma obtained from a Majorera male goat of the farm of the Las Palmas de Gran Canaria University was used. C3 was isolated as described for human C3 by Dodds [50] with minor modifications. Three chromatography steps were necessary: an anion exchange chromatography with a Hi Load 16/10 Q Sepharose FF column (GE Healthcare) with the buffers 20 mM HEPES, 50 mM ϵ-aminocaproic acid, 5 mM EDTA pH 7.4, plus 0.2 mM PMSF, and the same buffer plus 1 M NaCl; an anion exchange chromatography with a MonoQ HR 5/5 column with the same buffers; and finally, a gel filtration chromatography with a Superose 6 10/300 GL column in PBS-5 mM EDTA. Each step was analyzed with SDS-PAGE (9% (wt/vol) acrylamide).

#### SDS-PAGE

SDS-PAGE was performed using the method of Laemmli [56]. Sample buffer, sample preparation and staining with Coomassie Blue were as described by Fairbanks *et al.*[57]. All samples were run under reducing conditions.

Human C3 and human Factor H, used as standards in the gels, were purified as described in Dodds [50] and Sim *et al.*[55]. Human C1q was prepared as described by Tan *et al.*[58]. The molecular weight standard used was from Sigma.

## Authors’ contributions

IM carried out all the experimental work and manuscript preparation. AD coordinated and supervised C3 assays and manuscript preparation. AA and NC supervised and advised on scientific content of the manuscript and critical revision of the text. RBS designed, coordinated and supervised the study and manuscript preparation. All authors read and approved the final manuscript.
